# The Child as Econometrician: A Rational Model of Preference Understanding in Children

**DOI:** 10.1371/journal.pone.0092160

**Published:** 2014-03-25

**Authors:** Christopher G. Lucas, Thomas L. Griffiths, Fei Xu, Christine Fawcett, Alison Gopnik, Tamar Kushnir, Lori Markson, Jane Hu

**Affiliations:** 1 School of Informatics, University of Edinburgh, Edinburgh, United Kingdom; 2 Department of Psychology, University of California, Berkeley, California, United States of America; 3 Department of Psychology, Uppsala University, Uppsala, Sweden; 4 Department of Human Development, Cornell University, Ithaca, New York, United States of America; 5 Department of Psychology, Washington University in St. Louis, St. Louis, Missouri, United States of America; Brain and Spine Institute (ICM), France

## Abstract

Recent work has shown that young children can learn about preferences by observing the choices and emotional reactions of other people, but there is no unified account of how this learning occurs. We show that a rational model, built on ideas from economics and computer science, explains the behavior of children in several experiments, and offers new predictions as well. First, we demonstrate that when children use statistical information to learn about preferences, their inferences match the predictions of a simple econometric model. Next, we show that this same model can explain children's ability to learn that other people have preferences similar to or different from their own and use that knowledge to reason about the desirability of hidden objects. Finally, we use the model to explain a developmental shift in preference understanding.

## Introduction

A variety of studies [1]–[3] indicate that very young children make important inferences about the preferences and choices of others, a crucial part of the development of a “theory of mind”. However, the mechanisms that lead to such inferences are not clear. Developmental psychologists have suggested that children use evidence from their social environment to learn about preferences, but there has been no unified theory of how this learning occurs.

When learning about other people's preferences, adults rely on several kinds of information, ranging from overt expressions of pleasure or disgust, to subtler and less-direct information like the quantity and features of the options that the agent did not choose. Kushnir and colleagues [2] recently provided the first evidence that preschoolers can use also indirect cues, including the statistical properties of an agent's options, as the basis for understanding that agent's preferences. In another line of research, Fawcett and Markson [1] asked under what conditions children would use shared preferences between themselves and another agent as the basis for generalization. They found that children do not just use shared preferences as the basis for generalization, but also consider category membership. For example, given evidence that a person shares their preferences for specific toys, children are more likely to generalize a shared preference to novel toys than to novel foods. Finally, Repacholi and Gopnik [3] conducted an experiment to determine the age at which children come to understand that people have different preferences and act accordingly. They showed that 14-month-old children tend to offer other people the items that they themselves prefer rather than the items that those people have previously chosen, while 18-month-old children tend to make offers that reflect the past choices of the offer's recipient, suggesting that children come to understand preferences as person-specific mental states between those ages.

We present a rational model that explains these diverse results, and makes new predictions that have recently been tested empirically. Like other recent computational models of “theory of mind” development (e.g., [4], [5]), the model is based on the idea that children implicitly consider hypotheses that represent others' mental states or actions, and evaluate these hypotheses against data in accordance with Bayes' theorem. This model can be reduced to a set of commitments about the beliefs that children can entertain, the prior probabilities they implicitly assign to them, and how those beliefs connect to observable events. We propose that children assume that preferences are stable over time; that children can understand preferences as applying not just to individual objects, but to features or categories of objects; that children see preferences as varying in strength, with stronger preference for a feature leading to a greater probability of choosing options with that feature; and that children understand that choices can reflect both a preference for a chosen option and dislike for alternatives. While there are multiple ways to represent these commitments, we chose a specific model with origins in econometrics, the Mixed Multinomial Logit [6], for its simplicity and its widespread use in predicting choices in applied settings. The MML represents preference in terms of the subjective utility that different options provide the chooser, and assumes that choosers tend to make choices that maximize their utility. While people may not always make utility-maximizing choices in daily life, assuming that they do allows for a very good first pass at inferring their preferences, whether you are a child or a marketing researcher.

Our approach, realized through this model, provides a unified account of what might otherwise appear to be quite varied data across different studies, and accurately predicts new phenomena in preference learning. Moreover, as is always true with rational models, systematic deviations from the model are also informative about the processes underlying learning and the assumptions that children implicitly make.

## Model

Our general approach will be to consider how a child might optimally learn people's preferences from their choices, in the tradition of rational analysis [7]. A first step in such an analysis is defining a model of choice that captures children's assumptions about how people's preferences influence their actions. Given such a choice model, we can apply Bayes' rule to determine how an agent would make optimal inferences from others' behavior. Many such models are possible, but we will start by drawing from past research in psychology and economics that relates preferences and choices.

One of the simplest types of choice model asserts that, when faced with a set of options, people choose the one that they value most. In determining the values of options, people combine the values – or subjective utilities – of the features of those options, including some features that are only visible (or salient) to themselves. By imposing assumptions about how the utilities of these hidden features are distributed, one can specify a relationship between observable features, feature-specific utilities, and choice probabilities [8]. One of the most common assumptions is that hidden utilities follow a Gumbel distribution (or, in practice, a normal distribution [9]), which leads to a choice rule in which people are exponentially more likely to choose an option as its observable features become more attractive [10]. This simple choice rule is also commonplace in the psychological literature, where it has been called the Luce-Shepard choice rule [11], [12].

More formally, when presented with a set of 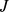 options with utilities 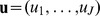, people will choose option *i* with probability proportional to 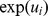, with
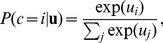
(1)where *j* ranges over the agent's options. Given this choice rule, learning about an agent's preferences is a matter of applying Bayes' rule. Specifically, given an observed sequence of choices 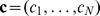, the posterior distribution over the utilities is:
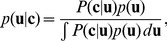
(2)where 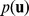 expresses the prior probability of a vector of utilities 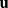. The likelihood 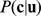 is the product of the probabilities of the individual choices given by Equation 1, assuming that the choices are independent given 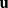. An option's utility is just the sum of the utilities of its features, so 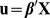, where 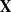 represents objects' features and 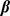 represents the agent's utilities for features. A final assumption is that 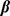 is normally distributed, with variance given by the parameter 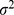.

This combination of prior and likelihood function – discussed at greater length in File S1 – corresponds to the Mixed Multinomial Logit model (MML; [6]), which has been used for several decades in econometrics to model discrete-choice preferences in populations of consumers. The MML and closely-related alternatives have been used to understand people's automobile ownership decisions and transportation choices [13], their decisions about telephone services and telephone use [14], and their choices of high- versus lower-efficiency refrigerators [15]. The MML's widespread application is due in part to the theoretical underpinnings of its choice model: the Luce-Shepard choice rule reflects the choice probabilities that result when agents seek to maximize their utility, making certain assumptions about the distributions over unobservable utilities [10], and is thus compatible with the standard assumptions of statistical decision theory. Our adoption of this model is driven in large part by its simplicity: given a minimal set of commitments about what preferences are likely – which we will detail later – we obtain a version of the MML that has few free parameters, in some cases just one, allowing us to compare model predictions to developmental data without being concerned that our fits are merely due to using a highly flexible model and choosing parameter values that happen to work.

## Results

The model outlined above provides a rational answer to the question of how to infer the preferences of an agent from his or her choices. In the remainder of the paper, we explore how well this answer accounts for the inferences that children make about preferences, applying it to the key developmental phenomena mentioned in the introduction as well as recent experiments explicitly designed to test its predictions. Our aim is not to provide an exact correspondence between model predictions and the available data, but rather to show that a rational model explains several phenomena with greater precision than do past accounts that only address subsets of the available data. For example, Kushnir et al. [2] argue that children use statistical information to distinguish between random and non-random patterns of choices, and use that information to learn about preferences. While that explanation is consistent with their data, our model makes more specific predictions about the patterns of children's judgments, explains generalization behavior in Fawcett & Markson's [1] results, and predicts inferences to graded preferences. Repacholi and Gopnik [3], in discussing their own results, suggest that children at 18 months see increasing evidence that their their caregivers' desires can conflict with their own. Our model is consistent with this explanation, but provides a specific account of how that evidence could produce a shift in inferences about new individuals. Details of how we obtained our predictions can be found in the Materials and Methods.

### Using statistical information to infer preferences

An experiment conducted by Kushnir et al. [2] provides evidence that children are sensitive to statistical information when inferring the preferences of agents. In this study, 3- and 4-year-old children saw one of three simple demonstrations. Each child was shown a box of toys, with the specific mixture of toys varying according to the experimental condition. In the 100% condition, the box contained just one type of toy (e.g., red discs). In the 50% condition, the box contained equal numbers of two types of toys (e.g., red discs and blue plastic flowers). In the 18% condition, the box contained two types of toys, but one toy was relatively rare (e.g., 18% red discs and 82% blue plastic flowers). A squirrel puppet, or “Squirrel”, was introduced to each child. In all three conditions, the puppet looked into the box and picked out five red discs. The experimenter then placed three toys in front of the child, including a red disc (the target), a blue plastic flower (the alternative), and a yellow cylinder (the distractor). The child was asked to select the object that Squirrel liked. The entire process was repeated using a different set of objects. The children selected the target (the red disc) 0.96, 1.29, and 1.67 times (out of 2) in the 100%, 50%, and 18% conditions, respectively, indicating that children used the statistics of the puppet's options to infer his preferences.

**Figure 1:**
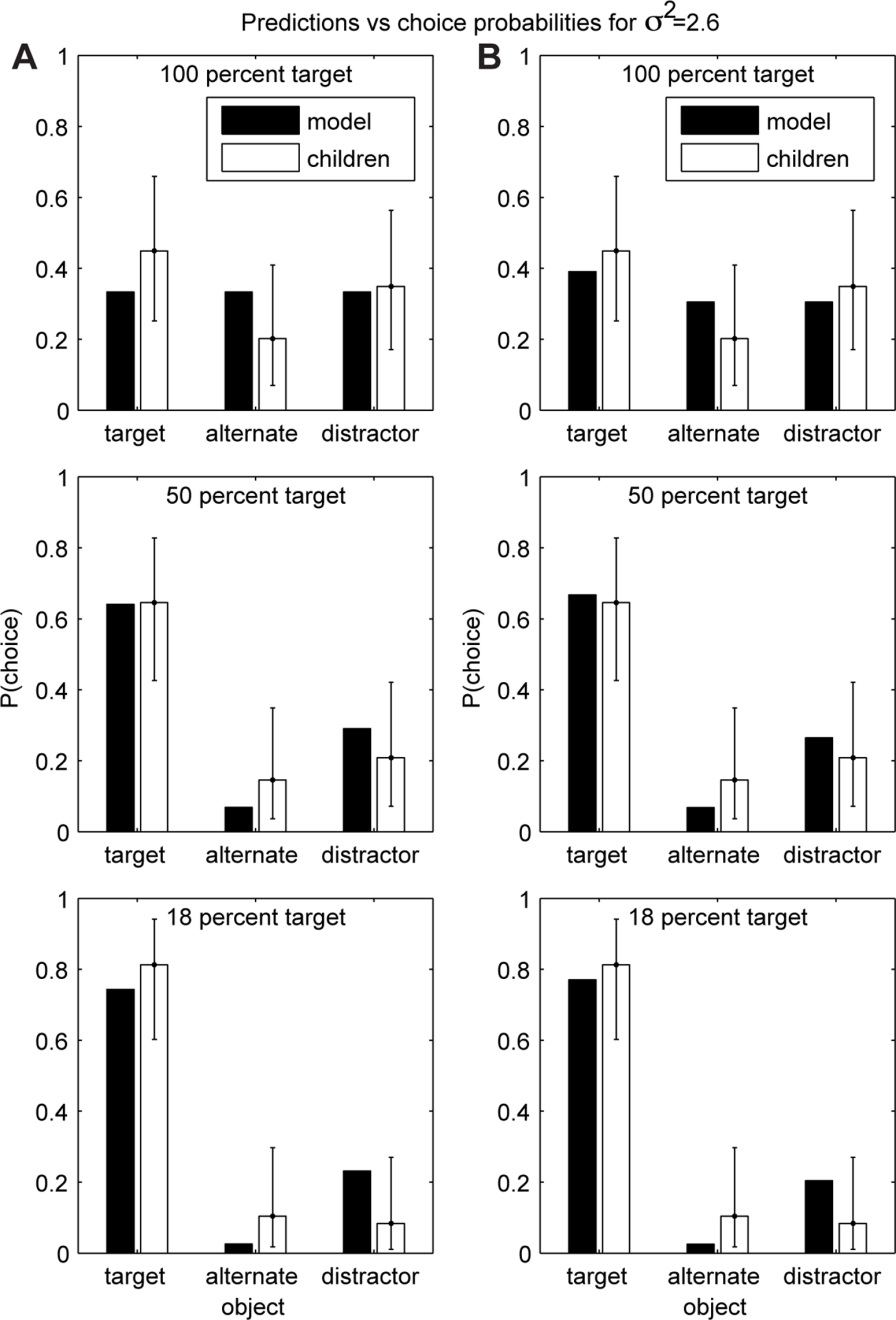
Model predictions and data for Kushnir, Xu, and Wellman's study [2]. (A) Predicted and observed proportions of children's offers under the default model. (B) Predicted and observed proportions of offers under the assumption that squirrel can decline to choose any object. Error bars represent 95% confidence intervals.

Figure 1(a) compares the predictions of the model to the children's offer frequencies. The model's mean squared error (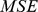) was .008 and the correlation between the model's predictions and mean child responses was 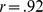. The model's only parameter is 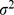, which has little influence on fits to the data (see File S1 for details). We found one notable difference between model's predictions and the children's choices: children tended to choose the target object more frequently than alternatives in the 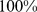 condition, while the model sees the 100% events as uninformative. While this mismatch may be an artifact – the difference between participants' choices and chance is not statistically significant – it also has a plausible explanation under our model: Squirrel could have done something other than select toys from the box, that is, he was choosing the target over other unobserved options. To test this idea, we included one other unobserved option at each choice event, with features orthogonal to the toys' features. The resulting predictions matched participants' offers more closely, yielding an 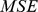 of 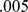 and a correlation of 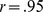. Figure 1(b) shows model predictions after this modification. If this explanation is true, it yields a new prediction: learners who see an agent making free choices should show a bias toward offering target object in the 100% condition, whereas in a control condition that makes it clear that the agent is required to choose something, that bias should disappear.

### Generalizing preferences to novel objects

Fawcett and Markson [1] went beyond asking children to learn preferences from choices, to exploring how two-year-old children solve the problem of using preference information to learn about novel hidden objects. Their experiments began with four training events, involving two actors (Actor 1 and Actor 2). At the start of every training event, each actor brought out an object, where both objects were members of the same category, e.g., food or toys. The actors displayed opposite preferences from each other, with each actor liking her own object and disliking the other object. Actor 1's objects were chosen to be consistently more interesting or desirable to the child. After each actor reacted to the objects, the child was given an opportunity to play with the objects, and his or her preference for one object over the other was judged by independent coders, based on relative interest in and play with each object. Following the training events, the children saw a test event in which each actor brought out an opaque container that hid an object. The hidden objects were said to belong to the same category as the training objects. Next, the actors reacted to the hidden objects in a manner that varied by condition. In the *positive* condition, each actor viewed the object and described it as her favorite member of the category. In the *negative* condition, each actor expressed dislike for her hidden object. In the *indifferent* condition, the actors did not see the new objects and professed ignorance about them. At the end of the test event, the child was then given an opportunity to choose one hidden object for him or herself. Finally, there was a second training event that differed from the first in one respect: the hidden objects were members of a different category from those seen in training. In Experiment 1, members of the new category were broadly similar to the training objects, e.g., books versus toys. In Experiment 2, the new category was intended to be quite different, e.g., food.

**Figure 2:**
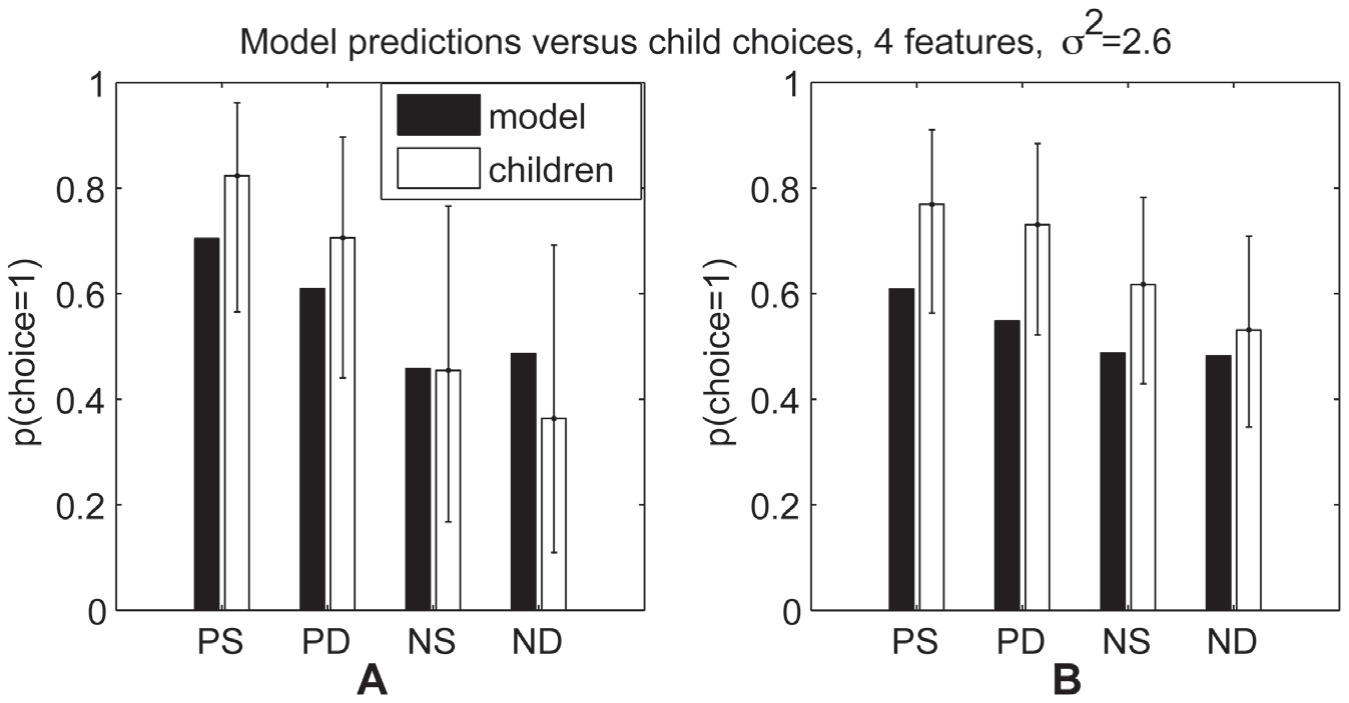
Model predictions for data in Experiment 1 of Fawcett and Markson [1]. (A) Results for children who showed a preference for 4 interesting toys. (B) Results for children who only showed a preference for 3 of 4 toys. The first character for each pair of bars denotes whether the actors showed a positive (P) reaction to the hidden toys versus a negative (N) reaction. The second character reflects whether the hidden object was said to be in a similar (S) or different (D) category from those seen in training. 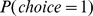 is the probability of selecting Actor 1's novel object. Error bars represent 95 percent confidence intervals. Cases where children had fewer than 4 chances to play with the training objects are excluded. For (A), there were 17, 17, 11 and 11 participants in the PS, PD, NS, and ND groups, respectively. For (B), there were 26, 26, 32, and 32 participants in the PS, PD, NS, and ND groups, respectively.

Figure 2 shows the MML's predictions and the rates at which children chose Actor 1's object. With 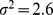 and twelve possible features, the correlation between the predictions and the overall choice rates was 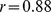. Predictive accuracy was generally insensitive to the number of features – with 30 features, the correlation dropped by only 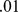. Children's choice proportions were more extreme than the probabilities predicted by the model, especially in the cases where children chose to play with one of Actor 2's objects during training. This could be because Actor 1's objects had features one would expect people to like a priori. Our model could accommodate this by using a non-zero mean for prior distribution on preferences. We could have achieved better fits by modifying the model to assign higher utilities to Actor 1's objects, thereby reflecting the a priori attractiveness of those objects, but that would have introduced an additional free parameter. Another unanticipated result is that there was a weak trend in the *negative* condition toward selecting Actor 1's objects when the novel items were different from the examples, versus similar. This can be seen in Figure 2, in which the *NS* judgments tend to favor Actor 1 more than the *ND* judgments. This difference did not significantly differ from the model's predictions or chance, and given the variability of the children's responses, might be due to (1) children choosing hidden object in service of gaining information about the experimenters' reactions rather than obtaining the more attractive option; or (2) failing to attend to the actors' emotional reactions, treating possession of the hidden objects as an implicit selection. Both of these possibilities may warrant further study.

### The developmental course of preference understanding

The next phenomenon we will consider is the developmental difference found by Repacholi and Gopnik [3], who compared 14- and 18-month-olds across two experimental conditions. In their *unmatched* condition, each child saw an actor express pleasure after tasting raw broccoli (which the children tended to dislike) and disgust after eating goldfish crackers (which the children tended to like). In the *matched* condition, the actor's pattern of reactions was reversed, matching the child's own. After presenting these reactions, the actor prompted the child to offer a food item by asking “Can you give me some?” and holding out a hand. In the *unmatched* condition, almost none (12.5 percent) of the younger children's offers matched the actor's previous choice of broccoli, while 69 percent of the older children's offers were broccoli. In the *matched* condition, where the actor chose the cracker, roughly equal proportions of offers by younger and older matched the actor's choice (72 percent and 75.9 percent, respectively). They found that between the ages of 14 and 18 months, children shift from offering actors the foods that the children themselves prefer to offering the foods that the actors previously selected. Repacholi and Gopnik offered the explanation that children see conflict in desire as evidence for preference differences. We will show that our approach provides a more precise version of their account, treating the developmental shift as the result of a rational interpretation of the evidence that young children are likely to observe. Given only a few observations, it may be rational for a child to believe that everyone's preferences are the same, or that “preferences” are merely recognition of the intrinsic goodness of the available options, even when more numerous observations with the same pattern support the belief that people have different preferences. Shifting from one model to another in this way is a consequence of the fact that simpler models tend to be more probable than more complex models with similar accuracy. Complex models, with larger numbers of parameters and the flexibility to explain a wide range of possibilities, assign probability to events not supported by observed data. Until enough events that are improbable under the simpler model are observed, the more complex one should be discounted. In the context of Bayesian model selection, this effect is called the Bayesian Occam's razor [16].

In the case of preferences, the simpler model (Model 1) assumes that all people have the same preferences, drawn from a normal distribution with mean zero. The more flexible model (Model 2) is the one we have been using: each person has a distinct set of preferences, which are drawn from the same distribution. If a learner sees choices made by people with distinct but similar preferences, the simpler model can explain a small number of choices well, as there is insufficient evidence to distinguish between noise and individual differences. As the number of observed choices grows, however, the simpler model will fail to account for the subtle but increasingly reliable differences between individuals, making it more and more likely that the flexible model is correct. We believe that most young children find themselves in a situation like this, because their preferences are broadly similar to those of their caregivers and siblings, and it may take quite some time to observe enough evidence to reveal individual differences.

**Figure 3:**
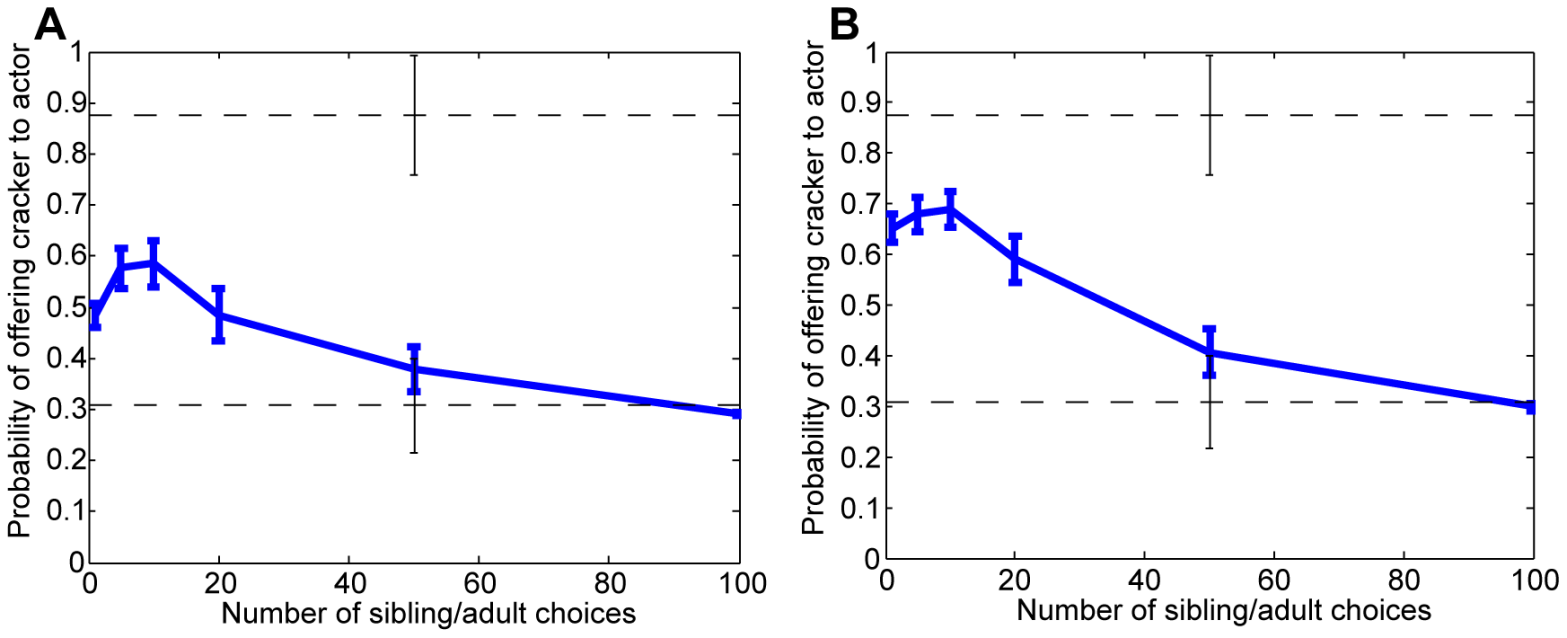
Results of simulations of the unmatched condition from Repacholi and Gopnik [3]. Each line shows the mean across 15 simulations, with standard errors. In both plots, the upper dashed line marks the proportion of 14-month-olds who offered the actor goldfish over broccoli (7 of 8), while the lower dashed line marks the proportion of 18-month-olds who did so (8 of 26), with standard errors. Plot (a) assumes equal prior belief in each model, while (b) assumes that the simpler model has a prior probability of 0.9.

As predicted by our simulations (described in the Materials and Methods), smaller amounts of data favor the simpler model, leading to the prediction that the actor has preferences like the child's and is likely to want a cracker. As data accumulate there is a shift toward the flexible model, leading to a higher probability that the actor wants the broccoli, because the flexible model treats the actor's choice of broccoli over goldfish crackers as the only event that is diagnostic of her preferences. The specific probabilities are given in Figure 3(a), assuming that both models are equally likely a priori.

The model makes another prediction: children who are in the process of shifting between the two views of preferences should be sensitive to the strength of evidence that the broccoli-choosing actor likes broccoli. Specifically, if a learner assigns non-negligible probability to both Model 1 and 2, then stronger evidence for a broccoli preference on the part of the mismatched actor, e.g., more broccoli choices or choices in the face of more alternatives, should lead to a stronger belief for a broccoli preference under Model 2 as well as somewhat more evidence that Model 2 is correct. In a study exploring this question, Ma and Xu [17] found just such an effect, using an experimental design similar to that used in Kushnir et al. [2]: 16-month-olds who saw an actor choose a boring object six times when there were more numerous exciting alternatives were more likely to later offer a boring toy over an exciting one (44 percent of cases) than were 16-month-olds who saw six choices where the boring toy was the only option (9 percent of cases).

One prediction that is not reflected in Repacholi and Gopnik's results is that the probability of offering the goldfish will rise initially, after a very small number of choice events, before falling again. To understand this, note that when only a handful of non-experimental choices have been observed, the events in the experiment constitute a significant proportion of the total evidence, which leads the flexible model to be favored. A possible explanation for the lack of evidence for such a trend is that children are predisposed to believe that the simpler model is more likely. Figure 3(b) shows the inferences that our simulations predict in the case where the simpler model is believed to be correct with a prior probability of 0.9. The resulting predictions are closer to the proportions seen in children's choices. Alternately, we may treat this difference as a new prediction that could be tested using a longitudinal replication of Repacholi and Gopnik's study.

### Testing new predictions: Learning graded preferences

**Figure 4:**
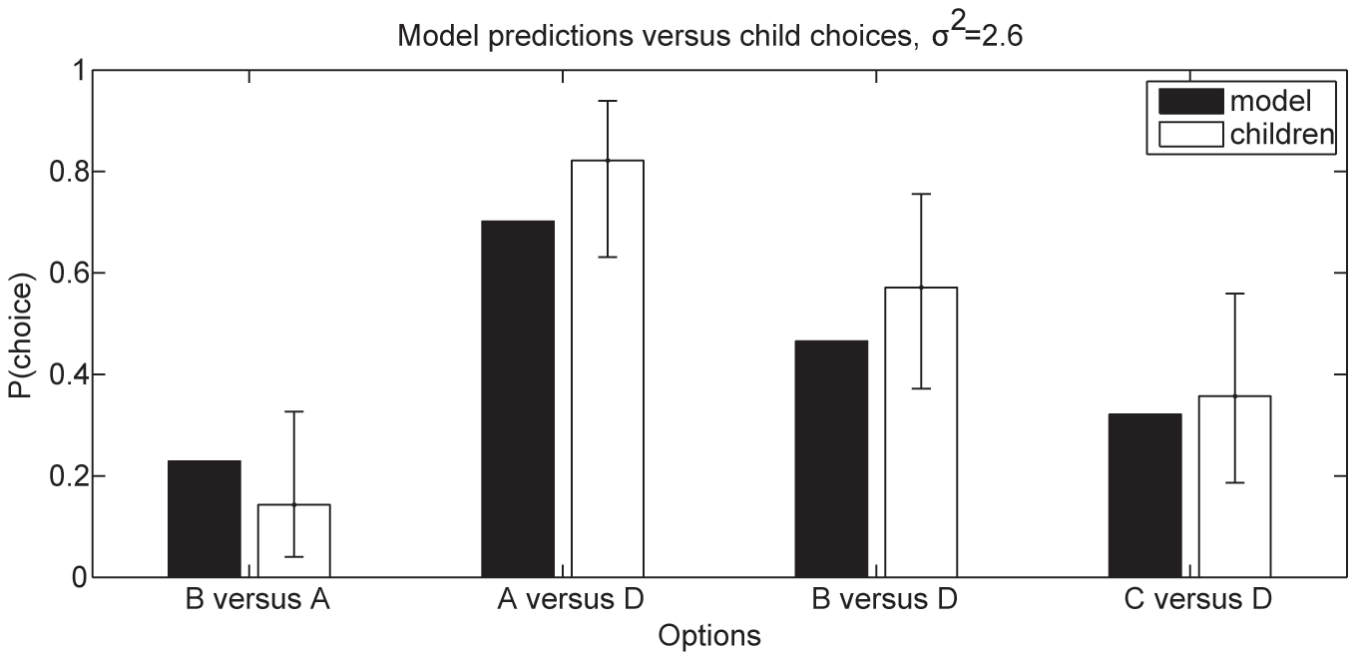
Model predictions for Hu et al.'s experiment. Predicted probability that objects will be selected, plotted against observed proportions, where A was chosen over C 7 of 10 times, B was chosen over C 5 of 5 times, and D was a novel alternative.

In explaining their own results, Kushnir et al. [2] proposed that children use statistical evidence to make a binary judgment of whether or not an individual prefers an object. In contrast, the MML model predicts that children are also sensitive to the strength of a person's preference. To test this prediction, Hu et al. (unpublished data; manuscript under revision) conducted two experiments studying 4-year-old children's inferences to graded preferences. In one experiment involving 31 preschoolers aged 44–63 months, children watched a puppet choose toy *A* over toy *C* five times. The puppet also chose between toys *B* and *C* 10 times, choosing toy *B* 7 of 10 times. Though objects *A* and *B* were never directly compared in the puppet's demonstrations, 86% of children successfully inferred that the puppet preferred toy *A* (chosen in 100% of the trials it appeared in) over toy *B* (chosen in 70% of the trials in which it appeared). When asked to compare objects *A*, *B*, and *C* to a novel object *D*, 82% of children inferred *A* would be preferred over *D*, 57% inferred *B* would be preferred over *D*, and only 36% inferred *C* would be preferred over *D*. Children's inferences suggest they used the consistency of the puppet's choices to determine the puppet's preferences (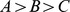), rather than the raw number of times each toy was chosen. The MML predicts this result (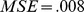, correlation 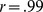 with choice proportions; see Figure 4) because a large number of choice events can provide compelling evidence that a preference exists, but only consistent choices provide evidence that an agent has a strong preference. More formally, numerous choices favoring an option can strongly indicate that its features have a positive subjective value, but the magnitude of that value depends on choice consistency.

## Discussion

Our goal has been to understand how children reason about the preferences of other people, and to explain their ability to learn from statistical evidence, generalize within and across categories, and discover that other people have their own distinct preferences. To that end, we used a model with roots in econometrics to see what inferences a Bayesian learner might make in these circumstances, making some simple assumptions about how preferences relate to choices. This model's predictions are consistent with children's judgments across a range of experimental conditions. In Kushnir et al. [2] and Fawcett and Markson [1], the model predicts children's sensitivity to the contexts of others' choices, their inferences from others' emotional responses, and their generalizations across categories. The model also shows how conceptual change in preference understanding is consistent with Bayesian inference, adding to a growing body of literature demonstrating that Bayesian methods provide elegant explanations for conceptual change [18]. We will next address some remaining issues, first discussing the appropriateness of describing the MML as a rational model, then assessing some alternative models of preference learning, and finally describing how our findings relate to children's theory of mind in general.

### Rationality in decision making and alternative models

In the view of preference learning that we have proposed, it is necessary to commit to a model of how preferences lead to choices, reflecting a set of assumptions on the part of the child. To the extent that ours is a rational analysis in the spirit of [7], those assumptions must reflect the true structure of the environment.

While the choice model used in the MML is not descriptively accurate under all conditions, we have found that it is largely indistinguishable from alternatives in the contexts we have considered, and that the most salient of these alternatives have disadvantages that preclude their use as the basis for a rational model, leaving the MML's choice model as the best available proxy for an ideal one. We tested an alternative approach based on Tversky's “Elimination by Aspects” (EBA) choice model [19], and found that a straightforward version could not account for many of the basic phenomena we observed. An extension of the EBA-based model, incorporating numerous hidden features and preferences, did not show these qualitative failures but still gave a worse account of our data than the simpler MML. See File S1 for details of these comparisons, as well as a discussion of other kinds of models.

While other choice models might be used in place of the MML, we do hold that several core assumptions of the MML are essential to any appropriate choice model: preferences are largely stable, though context-dependent factors might apply as well; preferences apply to choice categories or features, rather than just tokens; and preferences are graduated, with stronger preferences leading to higher choice probabilities.

### Further novel predictions

The MML makes additional predictions which we hope to test in future work. One prediction is that children can generalize preferences on the basis of specific features in addition to category membership: if an agent chooses diverse objects that are all red, then children should infer that red objects are desirable to that agent. A second prediction – which already has some support [17] – is that experience determines the age at which children understand that others have distinct preferences: children who observe more disagreements should pass Repacholi and Gopnik's task earlier. This suggests the possibility of leading children to earlier preference understanding with a training study. Developing new experiments to test these predictions will complement the work we have presented in this paper, providing a more complete evaluation of the model we have described and new ways to explore the richness of children's preference understanding.

### Modeling theory of mind

Before concluding, we will discuss how this work speaks to the development of theory of mind in general. Most work using probabilistic models has focused on children's understanding of physical causality, such as the action of blocks on machines. The work we have presented, along with that of Goodman et al. [5] and Seiver et al. [20], suggests that this kind of modeling can be equally effective in helping us understand children's developing knowledge of psychological causality. In particular, inferring preferences from choices underlies a wide range of more sophisticated understandings of the mind such as the inference of personality traits or intuitive judgments about the decisions of others. We know that even infants understand that human action is directed toward particular goals [21]. If children assume or learn representations of preferences like those in the MML model early in development, such assumptions could bootstrap a variety of sophisticated abilities to learn about the minds of others. Moreover, although much of the focus in the theory of mind literature has been on belief states, it may be more important, from an evolutionary point of view, for children to be able to infer the desires and preferences of others.

Our model highlights the question of how children represent the features of complex objects and events, which is a fundamental issue not just in theory of mind, but cognitive development more generally. Our results do not depend strongly on what features children use to represent options, as long as those features reflect inter- and intra-category similarity, but there might be cases where different feature choices lead to dramatically different inferences. For example, if an agent chooses options using a feature that is not salient to children, they might make spurious inferences about the attractiveness of other, correlated features. It is also possible that children use statistical regularities, both across options and others' choices, to determine what features to represent, in the vein of [22].

This project is intended to be a step towards a general account of theory of mind, one that addresses the human ability to learn about diverse mental attributes including beliefs and goals as well as preferences. With that aim in mind, it may be fruitful to explore the connections between our work and that of Baker et al. [4], [23], which explains how people infer goals and beliefs from sequences of actions and information about what an agent can observe. An extension to their model to represent preferences – via the MML – could explain a wide range of mental state attributions and the sources of information that drive them.

### Conclusion

Recent studies have shown that young children have a rich understanding of the relationship between preferences and choices. Not only do children think of other people as having their own idiosyncratic likes and dislikes, but children can learn about those preferences, not just from people's overt reactions to options, but from the contexts in which choices are made. Moreover, children can generalize preferences to new objects in a way that is sensitive to category membership, even when those new objects are hidden.

Taken together, this evidence provides a foundation on which to build a general account of preference learning. We have offered such an account, using a model borrowed from economics. It rests on the simple assumption that, in the mind of the learner, people pick options with the greatest subjective utility. This model explains children's talents in learning and generalizing from preferences, and shows that we can understand a developmental transition – in which children begin to recognize the idiosyncratic nature of preferences – as the result of a rational inference. In addition to explaining results from three separate papers and making predictions that are supported by a fourth, our model provides the first systematic approach to understanding preference learning in children, offers new predictions, and provides a bridge to other new research into children's theory of mind.

## Materials and Methods

### Ethics Statement

All of the studies described in this paper were approved by institutional review boards at the University of California, Berkeley or the University of Michigan. All participation was voluntary, with informed consent obtained from parents in writing.

### Predictions for Kushnir et al. and Hu et al

In Kushnir et al.'s experiments, children were asked to pick out the toy that Squirrel prefers, having observed that Squirrel chose a target object such as a red circle five times, from a pool of objects that included instances of the target object and an alternate object such as blue flowers. We can decompose this task into learning about Squirrel's preferences and using that knowledge to offer an object. Squirrel's choices reveal his preferences via their likelihoods: if his choices 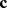 are much more likely given a strong preference 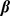 for the target object, then a strong preference is more probable, via Bayes' rule:
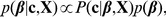
(3)where 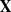 represents the options' features. In the 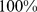 condition, where the target object constitutes all of Squirrel's options, Squirrel's preferences do not determine the likelihood of his choices – he must choose the target, regardless of what he likes – so no conclusions can be drawn from the choices the child sees. In the 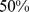 condition, the pattern of choices is more likely given a preference for the target object, because if Squirrel were indifferent to the different kinds of objects, he would choose one at random at each opportunity, so the likelihood of the actual events is 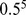, while a Squirrel with a strong preference should make those choices with high probability. This difference in likelihood is even more pronounced in the 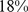 condition, where the probability that an indifferent Squirrel would choose the target object five times is 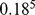. Note that indifference and strong preference are just two cases in the continuous range of preference that the MML can represent, and it assigns probabilities to all possible preferences over the observed objects or features.

Having learned about Squirrel's preferences, the child must now select an object to give Squirrel from a set consisting of one target object, one alternative object that was among Squirrel's options in the 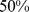 and 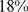 conditions, and one novel distractor object. If we suppose each child is choosing as Squirrel would, we can use the Luce-Shepard choice rule (Equation 1) to predict the rates at which children should choose the different objects for a particular set of preference values, and average over preference values to predict how often they should choose each item.

The same logic applies to Hu et al.'s studies, with each perceptually distinct object category having one distinct feature, and the numbers of options and observed choices matching those in Hu et al.'s experimental design.

### Predictions for Fawcett and Markson

In Fawcett and Markson's task, children selected hidden objects based on actors' expressions of dislike or declaring the object to be their favorite, the actors' earlier choices, and the category of the hidden objects. Actor 1 consistently chose attractive objects, Actor 2 consistently chose unattractive objects, and the category of the hidden objects was either similar to or different from that of the actors' earlier options. The generalization involved in this task requires two kinds of inferences. The first inference, to the actors' preferences based on their choices between the four pairs of boring and fun objects, is the same as the inference necessary in Kushnir et al.'s tasks. The second inference, to the hidden objects' features based on the actors' inferred preferences and their reactions, is somewhat different: rather than choosing objects, the actors gave emotional responses to them, and children had information about the object's category, constraining its possible features.

In applying our model to this task, we accepted the actors' statements at face value, taking expressions of dislike to mean an option's utility must be less than zero, and taking “my favorite” to mean an object must have the highest possible utility for its category. For the hidden objects' features, we assumed that each object has a set of category-specific features as well as features that span multiple categories. See File S1 for details.

The result of these two inferences is a distribution over the possible features of the two hidden objects, which we can translate into predictions about the child's choices once we know about the child's own preferences. Rather than making assumptions about the child's preferences, we use the child's choices over the original objects to infer his or her preference, again using the MML model. As discussed below, we might have used an informative prior (assuming children are more likely to prefer the interesting objects) to improve the fit of our model, but this would have come at the cost of introducing additional free parameters. See File S1 for details on both inference steps.

### Simulations for Repacholi and Gopnik

In modeling the developmental shift discovered by Repacholi and Gopnik, we assume that the child observes her own choices and those of her parent and a sibling. The preferences underlying those choices are given in Table S2 in File S1, as are the features we chose for the different food options, four of which are available at any choice event. We chose the options and features heuristically, with the aim that they be consistent with the preferences about foods exhibited by adults and children [24]. We do not assume that the child has direct access to her own preferences, but we account for the fact that she observes many more of her own choices than those of others by supposing she sees ten times as many of her own choices as choices by the other agents. The overall pattern of results is unchanged if we assume the child has direct knowledge of her own preferences (see File S1 for details).

Using these data, we can determine how a rational learner's predictions about a new broccoli-choosing actor's preferences should change over time: as the learner observes more choices, her adoption of a simpler model (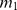) versus a more flexible model (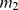) will change, as will her beliefs about what preferences agents should have under each model, leading to different predictions about the probability that the new agent should pick broccoli over goldfish, or vice versa. Formally, if 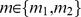 denotes the model, 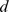 denotes the available data – choices observed along with agent identities and features of options – and 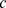 denotes the actor's next choice, then
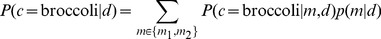
(4)where 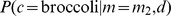 reflects only the new actor's previous broccoli selection because all agents have independent preferences under Model 2. In contrast, 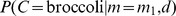 considers every choice event as if it had come from a single agent, so the probability of the actor choosing broccoli again will be dominated by the child's own preferences, which are responsible for most of the observed events. The posterior probability of model 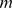, 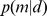, is proportional to 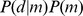 (see File S1 for details).

## Supporting Information

File S1 **Combined supporting information.** Contains a more detailed description of the MML model, details of its application to the experiments we have considered, and a discussion of alternatives to the Luce-Shepard choice model.
(PDF)
