# Supplementary material for: The Child as Econometrician: A Rational Model of Preference Understanding in Children
Source: PLoS One. 2014 Mar 25;9(3):e92160. doi: 10.1371/journal.pone.0092160 (PMC3965422; doi:10.1371/journal.pone.0092160)
Supplement: File S1 — Combined supporting information. Contains a more detailed description of the MML model, details of its application to the experiments we have considered, and a discussion of alternatives to the Luce-Shepard choice model. (PDF) [file pone.0092160.s001.pdf]

Supporting information for "The child as econometrician: A rational model of preference understanding in children"

This document contains details of the MML (Mixed Multinomial Logit) model, its application to the experiments in the main text, and alternatives to the Luce-Shepard choice model. Section 1 describes the central features of the model as it applies to all of the experiments. Sections 2, 3, and 4 detail the application of the model to the experiments of Kushnir et al. [6] and Hu et al. [4], Fawcett & Markson [2], and Repacholi & Gopnik [9], respectively. Section 5 addresses alternative choice models.

1 The mixed multinomial logit model

This section covers details of the MML. After describing basic properties of the MML as applied in this paper, it describes how predictions were generated for each of the three sets of developmental studies: Kushnir et al.'s and Hu et al.'s statistical preference learning studies; Fawcett and Markson's generalization results; and Repacholi and Gopnik's developmental transition.

A central feature of the MML is the choice rule that it assumes, namely that the probability of choosing an option increases exponentially with that option's utility, which is taken to be the sum of its features' utilities. In other words, if we let $\mathbf{x}_i$ be a binary vector indicating whether an option i possesses each of a set of features, and $\boldsymbol{\beta}$ be the utility that the agent of interest assigns to those features,[1] we can express the utility of option i for an agent as the inner product of these vectors. The probability of that agent choosing option i is then

$$P(c = i|\mathbf{X}, \boldsymbol{\beta}) = \frac{\exp(\boldsymbol{\beta}^T \mathbf{x}_i)}{\sum_j \exp(\boldsymbol{\beta}^T \mathbf{x}_j)}, \tag{1}$$

where $\mathbf{X}$ represents $\mathbf{x}_1, ..., \mathbf{x}_J$, the features of all of the options. We can also integrate out $\boldsymbol{\beta}$ to obtain the choice probabilities given just the features of the options, giving

$$P(c = j|\mathbf{X}) = \int \frac{\exp(\boldsymbol{\beta}^T \mathbf{x}_i)}{\sum_j \exp(\boldsymbol{\beta}^T \mathbf{x}_j)} p(\boldsymbol{\beta})\, d\boldsymbol{\beta}. \tag{2}$$

[1] We will refer to the utilities of features as "preferences" to distinguish them from the utilities of options.

While MML models can approximate the distribution of choices for essentially any population of utility-maximizing agents given appropriate choice of prior $p(\boldsymbol{\beta})$ [7], we use a common variant which supposes that $\boldsymbol{\beta}$ follows a Gaussian distribution around a population mean which itself has a Gaussian prior. In the case where no inferences are being made about average population-level preferences, this prior reduces to a single Gaussian with a fixed mean. We assume that mean to be zero for all features, consistent with the view that, in the absence of information to the contrary, a feature is unlikely to be very desirable or undesirable. More precisely, our prior is a Gaussian distribution with mean zero and variance σ^2: $\boldsymbol{\beta} \sim \mathcal{N}(0, \sigma^2\mathbf{I})$, so that preferences are independent across agents and features. The variance term σ^2 is the only free parameter.

2 Inferring preferences from statistical information

In order to make predictions for Kushnir et al.'s experiment, we first obtained a distribution over preferences given Squirrel's choices and options, and used those preferences to predict what object Squirrel would have chosen next.

Let $\boldsymbol{\beta}$ be Squirrel's preferences, $\mathbf{c} = (c_1 \dots c_N)$ the sequence of N choices Squirrel makes, and $\mathbf{X}_n = [\mathbf{x}_{n1} \dots \mathbf{x}_{nJ_n}]^T$ the observed features of Squirrel's J_n options at choice event n. The set $\{\mathbf{X}_1, \dots, \mathbf{X}_N\}$ will be denoted with $\mathbf{X}$. Estimating $\boldsymbol{\beta}$ entails computing $p(\boldsymbol{\beta}|\mathbf{c}, \mathbf{X}) \propto P(\mathbf{c}|\boldsymbol{\beta}, \mathbf{X})p(\boldsymbol{\beta})$, analogous to the inference to $\mathbf{u}$ in Equation 1 in the paper text. The probability of Squirrel's choices is $P(\mathbf{c}|\mathbf{X}, \boldsymbol{\beta}) = \prod_{n=1}^{N} P(c_n|\mathbf{X}_n, \boldsymbol{\beta})$, where $P(c_n = j|\mathbf{X}_n, \boldsymbol{\beta})$ is given by Equation 1.

We represented the objects using minimal and orthogonal feature vectors, so red discs (Squirrel's target toy) had features $[1\ 0\ 0]^T$, blue flowers (the alternative option in his choices) had features $[0\ 1\ 0]^T$, and yellow cylinders (the distractor) had features $[0\ 0\ 1]^T$, respectively. Each of the 38 objects was a separate option, where 38, 19, and 7 of the objects had features of the target object in the 100%, 50%, and 18% conditions, respectively, with the remainder having the features of the alternative object. The $N = 5$ choices made by Squirrel thus provide the data $\mathbf{c}$ from which its preferences can be inferred. We constructed an approximation to the posterior distribution over $\boldsymbol{\beta}$ given $\mathbf{c}$ using importance sampling (see, e.g., [8], for details), drawing 1E6 samples of $\boldsymbol{\beta}$ from the prior distribution $p(\boldsymbol{\beta})$ and giving each sample $\boldsymbol{\beta}^{(i)}$ weight w_i proportional to the corresponding likelihood $P(\mathbf{c}|\mathbf{X}, \boldsymbol{\beta}^{(i)})$.

The probability that the child will offer a particular item can be computed by assuming that the child matches the predicted probability that Squirrel would choose an object, integrating out $\boldsymbol{\beta}$:

$$P(c_{\text{child}} = j|\mathbf{X}, \mathbf{c}) = \int P(c_{\text{child}} = j|\mathbf{X}, \boldsymbol{\beta})p(\boldsymbol{\beta}|\mathbf{X}, \mathbf{c})\, d\boldsymbol{\beta}, \tag{3}$$

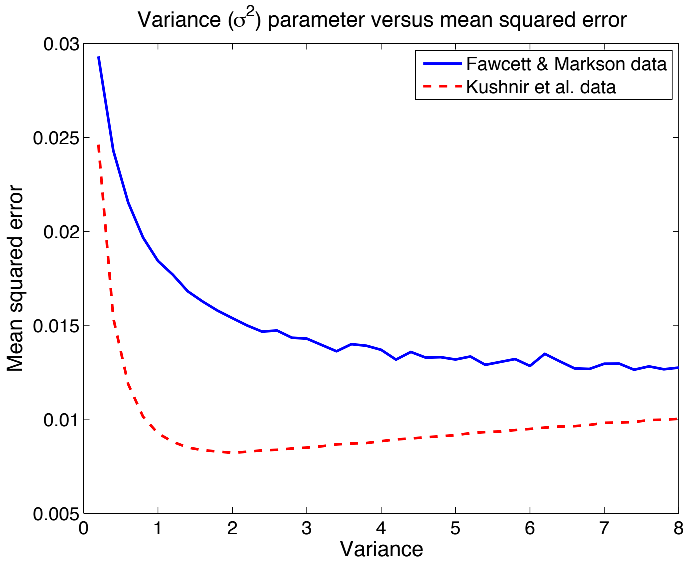

Figure S1: Sensitivity of model fits to setting of variance parameter, using data from Fawcett & Markson and Kushnir et al.

which is approximately equal to

$$\frac{1}{\sum_i w_i} \sum_i P(c_{\text{child}} = j | \mathbf{X}, \boldsymbol{\beta}^{(i)}) w_i. \tag{4}$$

Figure S1 shows that the goodness of fit is generally insensitive to the assumed variance of other people's preferences, σ^2, provided $\sigma^2 > 1$.

Note that model has two free parameters, including a variance term that determines the prior distribution over preferences, and the number of distinct features that each object possesses (one, in this case). Increasing the total number of features has the same effect as increasing the variance term: as the number of independent features increases, so does *a priori* absolute value of each option's utility.

3 Generalizing preferences to novel objects

As discussed in the main text, Fawcett et al.'s task involves two inferences. Formally, the first inference amounts to estimating $p(\boldsymbol{\beta}_a | \mathbf{X}, \mathbf{c})$ for $a = 1$ (for Actor 1, whose preferences match the child's) and $a = 2$ (for Actor 2, whose preferences differ), and it takes the same form as the preference learning described in the previous section.

Let the features of Actor a's preferred object in round n be $\mathbf{x}_{na}$, let the features of the same-category novel object be $\mathbf{x}_{sa}$, and let the features of the

different-category novel object be $\mathbf{x}_{da}$. When the category of a novel object is irrelevant, we will use $\mathbf{x}_{*a} \in \{\mathbf{x}_{sa}, \mathbf{x}_{da}\}$ to denote its features. The child's goal is to learn what features $\mathbf{x}_{*a}$ a novel object is likely to have, which permits her to know how much she will like each of the two novel objects and pick the better one. The available information includes the observed affective response of agent a, the features of the objects from the previous rounds $\mathbf{X}$, and the choices $\mathbf{c}$ of the agent on the previous rounds. Under our model, the distribution of a novel object's features given this information is

$$P(\mathbf{x}_{*a}|\mathbf{X}, \mathbf{c}, r_a) = \int P(\mathbf{x}_{*a}|\boldsymbol{\beta}_a, r_a) p(\boldsymbol{\beta}_a|\mathbf{X}, \mathbf{c})\, d\beta_a, \tag{5}$$

where $P(\mathbf{x}_{*a}|\boldsymbol{\beta}_a, r_a)$ is the posterior distribution over the features of the novel object given the preferences and affective response of the agent. Computing this distribution requires defining a likelihood $P(r_a|\mathbf{x}_{*a}, \boldsymbol{\beta})$ and a prior on features $P(\mathbf{x}_{*a})$. We deal with these problems in turn.

The likelihood $P(r_a|\mathbf{x}_{*a}, \boldsymbol{\beta}_a)$ reflects the probability of the agent producing a particular affective response given the properties of the object and the agent's preferences. In the experiment, the affective responses produced by the actors were of two types. In the *positive* condition, the actor declared that the hidden object was her favorite of the type. If one takes the actor's statement at face value and supposes that the actor has encountered an arbitrarily large number of such objects, then this favorite object has the most desirable possible combination of features given its category, possessing all category-appropriate features with positive utility, and no features with negative utility:

$$P(\mathbf{x}_{*a}|\boldsymbol{\beta}_a, r_a = \text{"positive"}) \propto 1 \text{ for } \mathbf{x}_{*a} = \arg\max_{\mathbf{x}_{*a}} \boldsymbol{\beta}_a^T \mathbf{x}_{*a}, \text{else } 0. \tag{6}$$

In the *negative* condition, the action – saying "there's a toy in here, but I don't like it"– communicates negative utility, or at least utility below some threshold we assume to be zero, so the object could be any member of the category with negative utility:

$$P(\mathbf{x}_{*a}|\boldsymbol{\beta}_a, r_a = \text{"negative"}) \propto 1 \text{ for } \boldsymbol{\beta}_a^T \mathbf{x}_{*a} < 0, \text{else } 0. \tag{7}$$

In defining the prior distributions from which the features of both the observed objects and the novel objects are sampled, it is important to represent differences between categories. Our feature vectors were concatenations of category-1 features, category-2 features, and multiple-category features, where each feature was present with probability 0.5 if its category could possess the feature, otherwise zero. We arbitrarily chose four features per category, for a total of twelve. As in the previous application of the model, increasing the number of features and increasing the variance term has the same basic effect on inferences, making them more extreme. It also was necessary to make an assumption about the extent to which different categories share features. This term influences how strongly or weakly inferences generalize across categories, and rather than fitting it, we chose a one-to-one ratio of intra- and inter-category features.

This combination of prior and likelihood function yields a posterior distribution over $\mathbf{x}_{*a}$, which can then be used to estimate the utilities of the hidden objects as a function of the child's own preferences. Unfortunately, we do not have direct access to the preferences of the children in this experiment, so we must estimate them from the children's choice data. We did this using the same procedure and priors as for the adults' utilities. That done, we apply the choice rule again to obtain choice probabilities for the novel objects selected by the children.

4 Developmental differences

To model the developmental shift described in Repacholi and Gopnik – their results are summarized in Table S1 – we conducted three sets of simulations. In the first set, which was designed to include interpretable and plausible features and options, we generated a series of choices by three agents: k choices by a parent, k choices by a sibling, and $10k$ choices by the child, with $k \in \{1, 5, 10, 20, 50\}$. Each choice was made from four options selected randomly without replacement from the set in the middle set of rows in Table S2. Two additional choices were appended to these data: one in which the child chose a goldfish cracker over broccoli, and one in which a new agent chose broccoli over the goldfish cracker.

The probability of offering the goldfish cracker to the actor was equal to the probability that the actor would choose the goldfish cracker herself, given by

$$P(c = \text{broccoli}|d) = \sum_{m \in \{m_1, m_2\}} P(c = \text{broccoli}|m, d)p(m|d). \tag{8}$$

The probability of model m_1 (same preferences) versus model m_2 (distinct preferences) is given by

$$P(m|d) = \frac{P(m)P(d|m)}{\sum_{m'} P(m')P(d|m')} \tag{9}$$

where d includes all of the available data, including choices $\mathbf{c}$, options and their features $\mathbf{X}$, and the identity of the agent making the choice, $\mathbf{a} = (a_1, a_2, ..., a_N)$.

Assuming that the identities of the agents and the available options are given,

$$P(d|m) = \int \prod_{i=1}^{N} P(c_i = j|\mathbf{X}, \boldsymbol{\beta}_a)p(\boldsymbol{\beta}_a|m, a_i)\, d\boldsymbol{\beta}_a. \tag{10}$$

If $m = m_2$, then a_i determines which agent's preferences are applicable at choice event i, while under m_1, the same preferences are used for all choice events, independent of a_i.

We generated predictions over fifteen separate runs, each with different, randomly selected options at each choice event. We used importance sampling to estimate the model and choice probabilities, drawing 2.5E4 samples per run per point in Figure 3 in the main text. For each sampling run, the high dimensionality of the space of preferences made it necessary to obtain proposal

Table S1: Summary of results from Repacholi and Gopnik, showing number of children in each condition (matched/unmatched) who offered broccoli, cracker, or neither.

Unmatched			
**Age**	**Cracker**	**Broccoli**	**Neither**
14-mo	7	1	34
18-mo	8	18	15

Matched			
**Age**	**Cracker**	**Broccoli**	**Neither**
14-mo	13	5	21
18-mo	22	7	8

distributions concentrated around the posterior, which we achieved by estimating preferences using 1.5E4 MCMC samples and generating proposals from a t-distribution fitted to those samples.

A second set of simulations using the same features demonstrated that the same effect occurs if the learner has direct access to his or her own preferences. In this case, the simpler model assumed that all individuals had preferences equal to the child's own. The more complex model used a prior centered on the child's own preferences – equivalent to combining the observed preferences with an uninformative prior – but did not assume that all agents' preferences are equal. Sixteen runs, using the same features and options as the first set of simulations, revealed a shift from predicting a goldfish preference for the new actor ($P(\text{goldfish}) = .67$) after 10 choice events involving a sibling or caregiver to predicting a preference for broccoli ($P(\text{goldfish}) = .47$) after 100 choice events.

We conducted a third set of simulations using a minimal set of features and objects, similar to those used in modeling Kushnir et al.'s results. There were three options, each with a single unique feature. The first option represented a goldfish cracker and the third represented broccoli. We represented three agents with preferences that all favored the cracker, but varied in their strength, shown in Table S3.

Because of the lower dimensionality of the preferences in Simulation 3, it was possible to use importance sampling alone to perform inference, using 3E6 samples per run. We conducted sixteen separate runs with all three options present at each choice event. We generated a series of k choices each agent, with $k \in \{2, 4, 8, 16, 32, 64, 128\}$. The results, shown in Figure S2, mirror those from the first two simulations.

5 Alternative choice models

In any rational analysis, a central assumption is that a learner's expectations are matched to the statistics of the environment [1]. As it applies to our analysis

Table S2: Preferences and features for objects used to establish a developmental transition in theories of preference in simulation of Repacholi and Gopnik, for Simulations 1 and 2.

	**Features**					
**Preferences**	fruit/vegetable	soft	sweet	liquid	milky	starchy
self	0	1	1.4	0.6	1.2	1.5
sibling	0.25	2	1.2	0.55	1.3	1.25
parent	0.5	3	1	0.5	1.4	1.0
**Options**						
raw vegetable	1	0	0	0	0	0
cooked vegetable	1	1	0	0	0	0
fruit	1	0	1	0	0	0
juice	1	0	1	1	0	0
dairy, not dessert	0	0	0	1	1	0
dairy, dessert	0	1	1	0	1	0
bread	0	1	0	0	0	1
cereal	0	0	1	0	0	1
desserts	0	1	1	0	0	1
soft drinks	0	0	1	1	0	0
broccoli	1	0	0	0	0	0
goldfish	0	0	0	0	0	1

Table S3: Preferences used to generate data for Simulation 3.

	**Features**		
**Preferences**	feature-1	feature-2	feature-3
Agent 1	3.0	1.5	0.0
Agent 2	2.0	1.0	0.0
Agent 3	1.0	0.5	0.0

Figure S2: Results of the third set of simulations showing an evidence-dependent shift in preference judgments. Each line shows the mean across 16 simulations, with standard errors. The upper dashed line marks the proportion of 14-month-olds who offered the actor goldfish over broccoli (7 of 8), while the lower dashed line marks the proportion of 18-month-olds who did so (8 of 26).

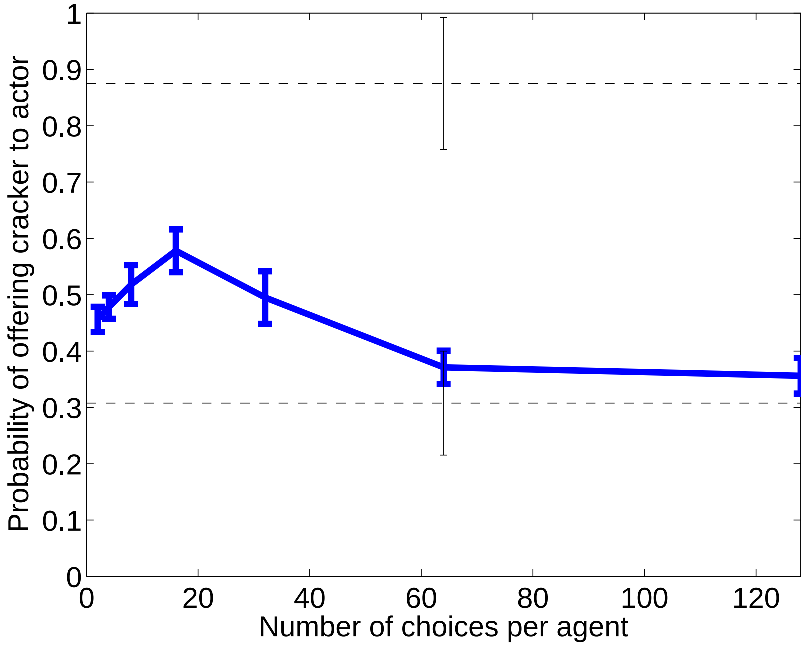

of preference learning, that assumption requires a choice model that accurately describes human choice behavior. The greatest challenge to the MML's choice model is that its assumption that people maximize their own stable, subjective utilities breaks down in the face of certain kinds of *decoy effects*. Specifically, introducing a "decoy" option with specific relationships with extant options can change people's choices [5, 12], a result that is not anticipated by the version of the MML that we have adopted.[2] A well-known case is the *attraction effect*: the MML predicts that introducing new alternatives will never make someone more likely to choose a particular option, yet when a new alternative is introduced that is *asymmetrically dominated*, or strictly worse than only one of two options, then people become more likely to pick the dominating option [5]. Other phenomena that cannot be explained by our specific model include the *similarity effect* [13], and the *compromise effect* [12], in which choice probabilities are influenced by new alternatives that are similar to one option or make one option appear to be a compromise between two extremes.

If we relax the assumption that other agents choose utility-maximizing options, it opens the door to a range of other models. Exploring such models may be useful in understanding what assumptions children and adults make about the factors that influence other people's decisions. One natural place to look for alternatives is the set of choice models that predict some or all of the decoy effects that frustrate the MML. If these models better capture the natural statistics of choices, they might be more suitable than the Luce-Shepard rule, leading to a "more" rational model of preference learning. One such choice model is Tversky et al.'s "elimination by aspects" (EBA) model of decision-making [13], which posits that people identify a desirable feature, discard options that lack that feature, and repeat the process with new features until only one option remains. This choice model can be used instead of the Luce-Shepard rule: a person's preferences under this model are feature weights, or the probabilities of using specific features to eliminate options. These weights are sufficient to estimate choice probabilities for a set of options, providing a likelihood function.

If we establish a prior over weights, then, as with the MML, Bayes' rule prescribes how preferences can be learned from choices. In its basic form, however, the EBA model fails to explain children's inferences in the studies we consider: Kushnir et al. found that children are sensitive to the number of alternatives rather than merely their observable features, while the EBA model is insensitive to counts of options – all options lacking a particular feature are eliminated at once, and additional same-feature items do not influence choice likelihoods.

It is possible to extend EBA-based models of preference learning to make them sensitive to option counts by adding random hidden features. This can be done by using a non-parametric Bayesian approach (e.g, [3]) – which makes inference intractable for the problems we consider – or treating the number of hidden features as an additional free parameter. We applied an EBA-based model to Kushnir et al.'s results, using both half-normal and Dirichlet priors

[2]This discussion will focus on instances of the MML using normal priors and no a priori assumptions about correlations between preferences. Given arbitrary priors, the MML can predict decoy effects, but at the expense of the simplicity we have emphasized.

on feature weights and between zero and forty hidden features, optimizing all parameters to fit the data. The best fits occurred with a Dirichlet prior with a concentration parameter of 0.15 and thirty hidden features, but these still gave a worse account of the data than the MML ($MSE = .02$ versus .008). We did not apply the EBA model to the other experiments because of its relatively poor performance in this case and the prohibitive computational cost of using it: whereas the MML has an analytic likelihood function, the EBA model's likelihood function required hundreds of samples to be drawn per likelihood value computed, and would have taken prohibitively long to generate predictions for Fawcett and Markson's data.

Other models been used to predict choice-making in the face of decoy effects, but it is unclear how they could be used to explain preference learning. To illustrate, we will consider two such models. The first, described by Roe, Busemeyer and Townsend [10], models the process by which choices are made, where valuations evolve over time until one option is chosen when its valuation crosses an inhibitory threshold. This model represents an individual's preferences using two sets of variables: valuations for different features of the available options, and variables that determine the bias and temporal dynamics of attention to different features. In principle, one could define priors over these parameters, determine a likelihood function for choices given their values, and use Bayesian inference to estimate the probable values for a given individual. The problem in inverting this model to recover preferences lies in its temporal aspect: a single choice emerges after a potentially large number of iterations in the model, with the consequence that it is extremely time-consuming to accurately determine the likelihood of a given choice. This problem is compounded by the large number of parameters, which includes a matrix of feedback weights and more in addition to the $(|\text{options}| + 1)|\text{features}|$ parameters that capture valuations and weights. It may be possible to develop heuristic approximations for choice likelihoods that are broadly consistent with this model, but in that event one might do better to build a heuristic model of preference learning directly. A second choice model, developed by Schneider, Oppenheimer, and Detre [11], accounts for decoy effects by assuming that people make choices in a process by which options are scored according to their rankings relative to distinct objectives, such as minimizing risk or maximizing expected value. While it offers a parsimonious explanation of decoy effects, Schneider et al.'s model does not include any specification of how individual preferences shape a person's choices, and thus cannot be used to develop a model of preference learning.

References

[1] J. R. Anderson. *The adaptive character of thought.* Erlbaum, Hillsdale, NJ, 1990.

[2] C. Fawcett and L. Markson. Children reason about shared preferences. *Developmental psychology*, 46(2):299, 2010.

[3] D. Gorur, F. Jakel, and C.E. Rasmussen. A choice model with infinitely many latent features. In *Proceedings of the 23rd international conference on Machine learning*, pages 361–368. ACM, 2006.

[4] J. Hu, C. Lucas, T. Griffiths, and F. Xu. Preschoolers' understanding of graded preferences. *Cognitive Development*, submitted.

[5] J. Huber, J.W. Payne, and C. Puto. Adding asymmetrically dominated alternatives: Violations of regularity and the similarity hypothesis. *The Journal of Consumer Research*, 9(1):90–98, 1982.

[6] T. Kushnir, F. Xu, and H. Wellman. Young children use statistical sampling to infer the preferences of others. *Psychological Science*, 21:1134–1140, 2010.

[7] D. McFadden and K. Train. Mixed MNL models of discrete response. *Journal of Applied Econometrics*, 15:447–470, 2000.

[8] R. M. Neal. Probabilistic inference using Markov chain Monte Carlo methods. Technical Report CRG-TR-93-1, University of Toronto, 1993.

[9] B. M. Repacholi and A. Gopnik. Early reasoning about desires: Evidence from 14- and 18-month-olds. *Developmental Psychology*, 33(1):12–21, 1997.

[10] R.M. Roe, J.R. Busemeyer, and J.T. Townsend. Multialternative decision field theory: A dynamic connectionst model of decision making. *Psychological Review*, 108(2):370, 2001.

[11] A. Schneider, D. Oppenheimer, and G. Detre. Application of voting geometry to multialternative choice. In *Proceedings of the 29th annual conference of the Cognitive Science Society*, pages 635–640, 2007.

[12] I. Simonson. Choice based on reasons: The case of attraction and compromise effects. *The Journal of Consumer Research*, 16(2):158–174, 1989.

[13] A. Tversky. Elimination by aspects: A theory of choice. *Psychological review*, 79(4):281, 1972.
